# Supplementary material for: Primary thermometry triad at 6 mK in mesoscopic circuits
Source: Nat Commun. 2016 Sep 23;7:12908. doi: 10.1038/ncomms12908 (PMC5036159; doi:10.1038/ncomms12908)
Supplement: Supplementary Information — Supplementary Figures 1-3 and Supplementary Notes 1-2 [file ncomms12908-s1.pdf]

I. SUPPLEMENTARY FIGURES

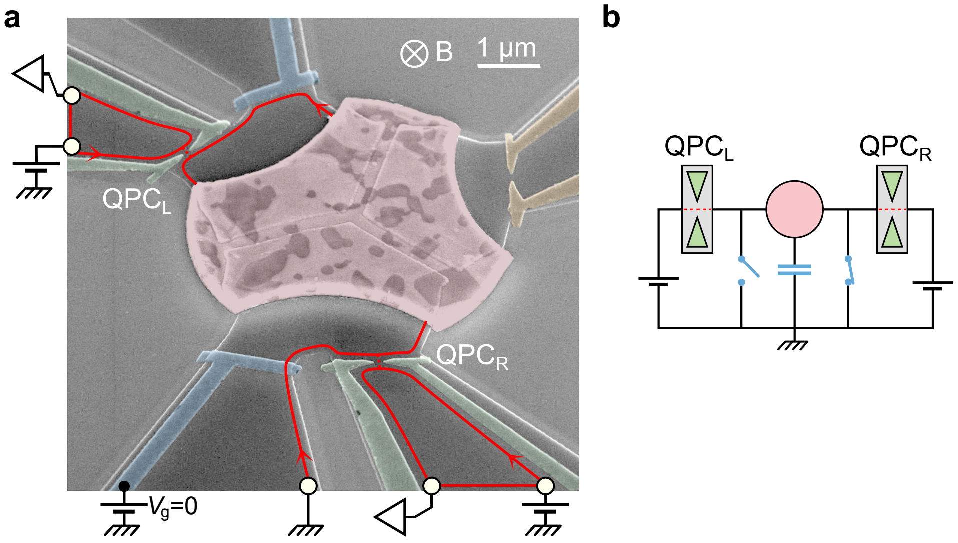

Supplementary Figure 1. **Quantum shot-noise configuration**. (**a**) Colored micrograph of the measured device. The displayed edge currents (red lines) here correspond to the circuit configuration used for the quantum shot noise measurements across QPC$_R$. The right short-circuit switch is closed by applying $V_g = 0$. (**b**) Corresponding circuit schematic.

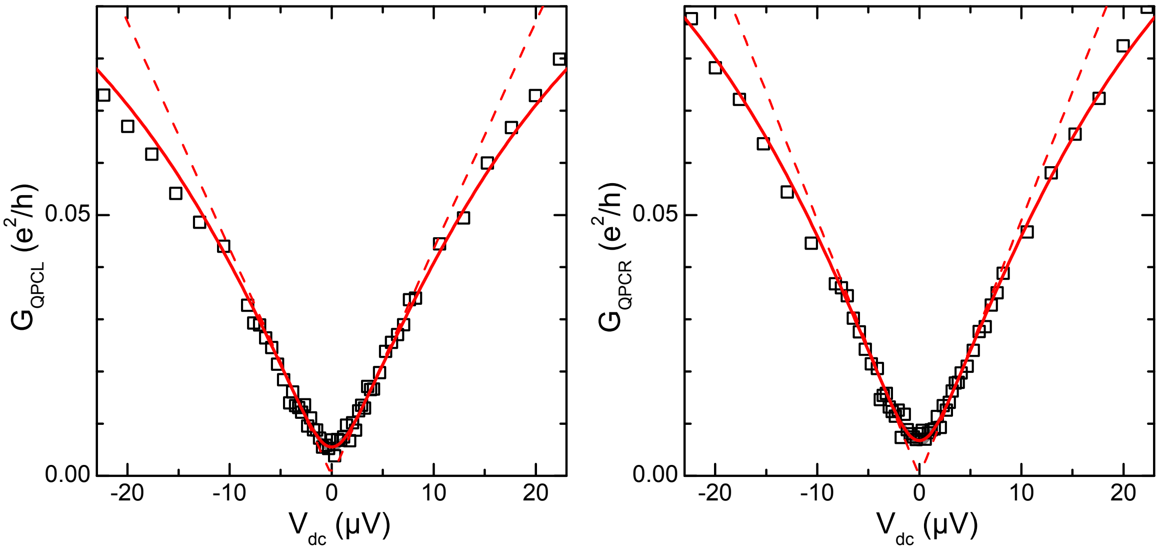

Supplementary Figure 2. **Dynamical Coulomb blockade data vs predictions**. The same data (symbols) and theoretical predictions (continuous lines: numerical calculations of the full prediction; dashed lines: $T = 0$ predictions at $eV_{\rm dc} \ll E_C$ of Eq. 4) shown in Fig. 2c are here displayed up to larger dc bias voltages.

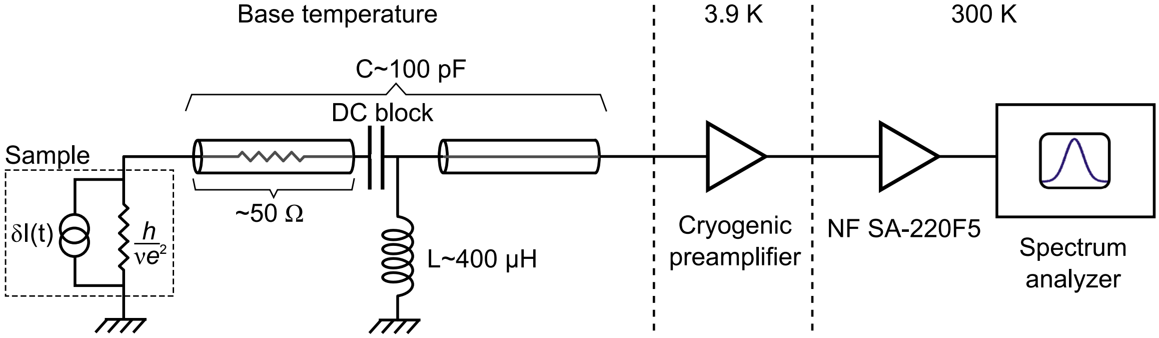

Supplementary Figure 3. **Noise measurement setup**. The current fluctuations $\delta I(t)$ are converted into voltage fluctuations through the resonator impedance Z consisting on the on-chip quantum Hall resistance $R = h/\nu e^2$, with ν the filling factor ($\nu = 6$ for the magnetic field $B = 1.4$ T) in parallel with a LC tank. The latter consists of a superconducting inductance $L \sim 400$ μH and the capacitance $C \sim 100$ pF developing along the coaxial lines resulting in a resonant frequency of $f_{\rm LC} \simeq 0.84$ MHz. The bandwidth at -3 dB of the overall resonator Z is equal to $1/2\pi RC$ and ranges from 370 to 120 kHz for $\nu = 6$ to 2. The achieved relative precision of the voltage fluctuation measurement can be increased by two parameters: the integration time t_i and for a white noise, the frequency interval Δf on which the measurement is performed. Indeed, the relative precision scales as $1/\sqrt{N}$ with N the number of measured samples, where N is proportional to the product of t_i and Δf. In practice, at $\nu = 6$ and $T = 6$ mK, we used $t_i = 40$ s per point and the frequency window $\Delta f = [0.68, 1]$ MHz that roughly matches the resonator bandwidth. Moreover, note that the data shown in Fig. 2a are an average of 131 sweeps, each consisting of 31 points, and were measured in about 2 days. The electronic temperature can be extracted directly, without calibration of the noise measurement setup, from the integrated raw signal.

II. SUPPLEMENTARY NOTES

Supplementary Note 1: Low temperature components of the experimental setup

Sample installation.

The measured sample is glued to the grounded gold back-plane of a ceramic leadless chip carrier (Kyocera, part number: PB-44713) and electrically connected by aluminum wire bonding.

The ceramic chip carrier is then plugged in a plastic socket (E-Tec, part number: LCC-044-H210-55) inside the top-loaded sample holder. The socket, reinforced laterally with epoxy resin (Stycast 2850FT with catalyst 9) and at the bottom with a stainless steel plate, is permanently screwed to the top-loaded sample holder.

The inner stainless steel shield, whose bottom inside surface is covered by a thin layer of microwave absorber (Eccosorb CR-124 epoxy resin), and the outer gold plated brass shield are screwed into position (see Fig. 4b).

The sample holder is then inserted from the top of our dilution refrigerator (Triton 200 from Oxford instruments), through rotating radiation shields, and screwed to the mixing chamber (Triton top loading option).

Measurement lines filtering and thermalization at low temperature.

The high-frequency filtering and initial thermalization is done following Ref. 20, using resistive microcoaxial cables. For each measurement line, one meter of a narrow resistive NiCrAlSi (Isaohm) wire (diameter 75 μm, resistance 300 Ω/m) is inserted into a CuNi tube of inner diameter 260 μm. The CuNi tubes are tightly coiled on a copper plate screwed to the mixing chamber of the dilution refrigerator.

The low frequency filtering is performed inside the top-loaded sample holder, using simple RC-filters with CMS components (nichrome resistances from Vishay TNPW series, C0G ceramic capacitors from Murata GRM series).

Additional high-frequency filtering is provided by $\sim$ 30 cm long CuNi microcoaxes (Coax Co. Ltd., part number: SC-040/50-CN-CN) between RC-filters and the inner stainless steel shield.

Inside the inner stainless steel shield, the strongest thermal anchoring of each measurement line is realized by dipping $\sim$ 5 cm of a copper wire coated with a thin insulating layer into a conductive silver epoxy (Epotek, part number: H20E) together with a thermalized copper braid.

Supplementary Note 2: Noise measurement setup.

The current fluctuations measurements are performed using a setup very similar to that described in more details in the supplementary material of Ref. 11, here implemented in a cryogen-free dilution refrigerator.

A schematic representation of the amplification chain for the current fluctuations measurement is shown in Supplementary Fig. 3. The current fluctuations are converted on-chip into voltage fluctuations using the well-defined quantum Hall resistance $R = h/\nu e^2$, with ν the filling factor ($\nu = 6$ at $B \simeq 1.4$ T). The most crucial element of the amplification chain is the home-made cryogenic voltage preamplifier (see Refs. [11,32,33]), which is thermalized to the 3.9 K plate. It is operated slightly below 1 MHz, where it shows the best performances and where the electrical noise induced by the pulse tube vibrations is found negligible. For this purpose, we shift the experimental frequency bandwidth with a parallel L–C tank circuit of resonant frequency $f_{\text{res}} \simeq 0.84$ MHz. The capacitance $C \sim 100$ pF is the capacitance that develops along the coaxial lines connecting the sample to the cryogenic preamplifier, whereas the inductance $L \sim 400$ μH is realized with a superconducting coil thermally anchored at base temperature but located away from the magnetic field. The current fluctuations signal remains within the same frequency bandwidth $\sim 1/2\pi RC$, now around f_{res}. Note that we use a dc block to ascertain that the amplification chain is not influenced by the dc voltage bias across the sample.

The spectral density of the measured current fluctuations is integrated over a frequency window that is optimized separately for each value of ν. At $\nu = 6$ ($B \simeq 1.4$ T), where we obtain the lowest electronic temperature $T \simeq 6.0$ mK, the optimized integration window is $f \in [0.68, 1]$ MHz. As described below, this raw integrated signal S_I^{raw} versus the dc bias voltage V_{dc} applied to the QPC can be used directly to extract the electronic temperature, without calibration of the noise measurement setup and without the knowledge of the transmission probabilities $\{\tau_n\}$ across the QPC.

More precisely, the excess raw integrated signal is simply proportional to the excess spectral density $\Delta S_I^{\text{raw}} \equiv S_I^{\text{raw}} - S_I^{\text{raw}}(V_{\text{dc}} = 0) = G\Delta S_I$, with G the effective amplification chain gain (depending on the frequency window). The excess raw integrated signal can then be fitted using Eq. 1 with two free parameters: the electronic temperature T and the product $G\sum \tau_n(1-\tau_n)$. The product $G\sum \tau_n(1-\tau_n)$ is given by the temperature-independent linear slope of $\Delta S_I^{\text{raw}}(V_{\text{dc}})$ at large $|V_{\text{dc}}| \gg k_B T/e$, and the temperature T is obtained from the zero to large voltage bias crossover. In practice, we start directly with the full (not excess) raw integrated signal and therefore also use the zero bias offset $S_I^{\text{raw}}(V_{\text{dc}} = 0)$ as a fit parameter.

With the additional knowledge of the transmission probabilities $\{\tau_n\}$ across the QPC, the effective amplification chain gain G can be extracted from the fit parameter $G\sum \tau_n(1-\tau_n)$. Although not necessary to extract the temperature T, such a calibration is used in the top panel of Fig. 2a in order to display the measured excess noise ΔS_I in units of A^2/Hz. Note that in practice the QPC is set to a single half transmitted channel, whose precise transmission probability $\tau \simeq 0.55$ is measured simultaneously to the current fluctuations.
